# Bone and Lean Mass Loss and Cognitive Impairment for Healthy Elder Adults: Analysis of the Nutrition and Health Survey in Taiwan 2013–2016 and a Validation Study With Structural Equation Modeling

**DOI:** 10.3389/fnut.2021.747877

**Published:** 2021-10-13

**Authors:** Sheng-Feng Lin, Yen-Chun Fan, Wen-Harn Pan, Chyi-Huey Bai

**Affiliations:** ^1^Department of Public Health, School of Medicine, College of Medicine, Taipei Medical University, Taipei, Taiwan; ^2^School of Public Health, College of Public Health, Taipei Medical University, Taipei, Taiwan; ^3^Department of Emergency Medicine, Taipei Medical University Hospital, Taipei, Taiwan; ^4^Department of Critical Care Medicine, Taipei Medical University Hospital, Taipei, Taiwan; ^5^Institute of Biomedical Sciences, Academia Sinica, Taipei, Taiwan; ^6^Nutrition Research Center, Taipei Medical University Hospital, Taipei, Taiwan

**Keywords:** bone loss, bone marrow density, cognitive impairment, dementia, osteoporosis

## Abstract

**Purpose:** Bone and lean mass loss and cognitive impairment are prevalent in elder adults and have been hypothesized to share a potential link.

**Methods:** This nationwide cross-sectional study systemically sampled elder adults aged ≥65 years and conducted the door-to-door survey. The causal diagrams help to decide which covariates were included in the generalized linear mixed models (GLMMs). The structural equation modeling (SEM) was performed for the validation.

**Results:** A total of 535 participants were enrolled and categorized into the normal (67.3%), mild cognitive impairment (18.3%), and dementia groups (14.4%). With increasing in the severity of cognitive impairment, the bone marrow density and lean mass consistently showed the trend of decreasing values. In the GLMMs, a significant association existed between the decrease of the bone mineral density (BMD) and the Mini-Mental State Examination (MMSE) (β = 5.819 scores per g/cm^2^ decrease, *p* = 0.0305) with adjustment of the age, sex, and physical activity. The SEM models confirmed that the MMSE was significantly and directly predicted by the age (β = 0.1363, *p* = 0.0003) and BMD (β = 0.1251, *p* = 0.0006) independently and indirectly predicted by lean mass (β = 0.1138, *p* = 0.0003) through the bone density path.

**Conclusion:** In conclusion, an independent association between bone loss and cognitive impairment was existed rather than the confounding effect and the decrease of lean mass indirectly contributed to cognitive impairment by influencing the bone density.

## Introduction

Skeletal deficit and muscle loss have emerged as major issues for elder adults (1–3). Several reports have documented the comorbidity of osteopenia and osteoporosis in patients with dementia (4–6). Bone loss has also been reported for sharing a distinctive connection to cognitive impairment (7). The mechanical unloading of the skeleton has been proposed to cause loss of bone mass in patients with aging (8). In addition, decreased physical activity (9), fragility, and sarcopenia (10) may also directly or indirectly contribute to bone loss in elder patients with dementia. Collectively, the connection between the boss loss and cognitive impairment is not easily elucidated and possibly as a result of confounding by related various factors.

Physiologists proposed a paradigm that bone remodeling and energy metabolism are coregulated through the brain–bone axis (11–13). The skeleton is a metabolically active system and undergoes bone resorption and bone formation in whole life (14). Investigation into the brain–bone axis began with an emphasis on leptin (15), a hormone secreted by the adipose cells with remarkable effects in the brain for coregulation of the appetite and bone accrual (12, 16). Low levels of leptin have been reported in Alzheimer's disease (16, 17). Moreover, the lean mass has been found in a major source of neurotrophic factors for preventing cognitive impairment (18). Accordingly, two correlated factors of bone and lean mass are needed to be considered simultaneously to explore the relationship between body composition and cognitive impairment in a statistical model. However, most of the epidemiological studies (4, 19–27) only considered one of the two correlated factors at a time. An epidemiological study investigating the link between the bone and lean mass cognitive impairment in elder adults, while effectively controlling multiple contributing factors is warranted.

In statistical analysis, one of the major challenges to investigating the influential factors on cognitive impairment is that the numerous variables, including age, physical activity, bone mass, and lean mass, are correlated with each other. With respect to the methodological advances and software development, a structural equation modeling (SEM) permits the illustration of the relationship among many factors (28, 29). The flexibility of SEM allows its application in a cross-sectional study and other research designs (28). While the causal relationship cannot be obtained from a cross-sectional study, a directed acyclic graph (DAG) provides a simple way to demonstrate the relationships between the variables and to evaluate if confounding was present in the model (30).

The National Nutrition and Health Survey in Taiwan (NAHSIT) 2005–2008 described that osteoporosis was estimated to affect one-fourth of the general population in Taiwan (31). A recent 2018 report indicated that the incidence and prevalence of osteoporosis in Taiwan were similar to those in most of the Western countries with aging populations as well (32). In this study, we like to investigate whether an association exists between cognitive impairment and bone loss in our community-based elder participants by using the latest survey data NAHSIT 2013–2016. We adopted two novel approaches: (1) using DAGs to identify the confounding variables that are needed to be adjusted in the conventional regression analysis and (2) conducting an SEM to validify the best model constructed by a DAG.

## Methods

### Study Design and Data Collection

The nationwide cross-sectional data were collected through the NAHSIT from January 1, 2013, to December 31, 2017, covering 359 townships or the city districts in Taiwan. The systematic sampling of the participants was classified into the eight strata by the characteristics of the population density, geographical area, and dietary habits. The door-to-door visits were carried out to obtain information of age, sex, body mass index (BMI), and physical activity by the trained interviewers. Mobile dual-energy X-ray absorptiometry (DXA) was performed to obtain the body composition parameters and the bone mineral density (BMD) in each specific body region. Based on the WHO definition, we defined osteoporosis as a BMD T-score (in g/cm^2^) of ≤2.5 at the femoral neck or the lumbar spine in those aged 65 years and older. The DXA device (Prodigy, GE Healthcare Lunar, Wisconsin, USA) was used. Elder participants aged ≥65 years who agreed to complete the physical assessment were enrolled. Besides, the lipid profiles, vitamin A, and vitamin D were measured in the centralized laboratory. This study was approved by the Institutional Review Board on Biomedical Science Research, Academia Sinica, Taiwan (AS-IRB01-13067) and the Research Ethics Committee, National Health Research Institutes, Taiwan (EC1020110). Informed consent was acquired from the participants.

### Cognitive Assessment

The diagnosis for dementia due to all the causes was following the guideline by the National Institute on Aging-Alzheimer's Association workgroups. Taiwanese version of the Mini-Mental State Examination (MMSE) (33) assessment was performed by the trained interviewers. The participants were categorized into the normal cognition, mild cognitive impairment (MCI), and dementia groups according to the previous literature. First, the participants with the MMSE scores of 27–30 (≥9 education years) and of 26–30 (<9 education years) were classified into normal cognition. Second, the participants with the MMSE scores of 24–26 (≥9 education years) and of 23–25 (<9 education years) were classified into MCI. Third, the participants with the MMSE scores of 14–23 (≥9 education years) and of 11–22 (<9 education years) were classified into moderate dementia. Finally, the participants with the MMSE scores of 0–13 (≥9 education years) and of 0–10 (<9 education years) were classified into severe dementia (33).

### Causal Diagram

**Figure 1:**
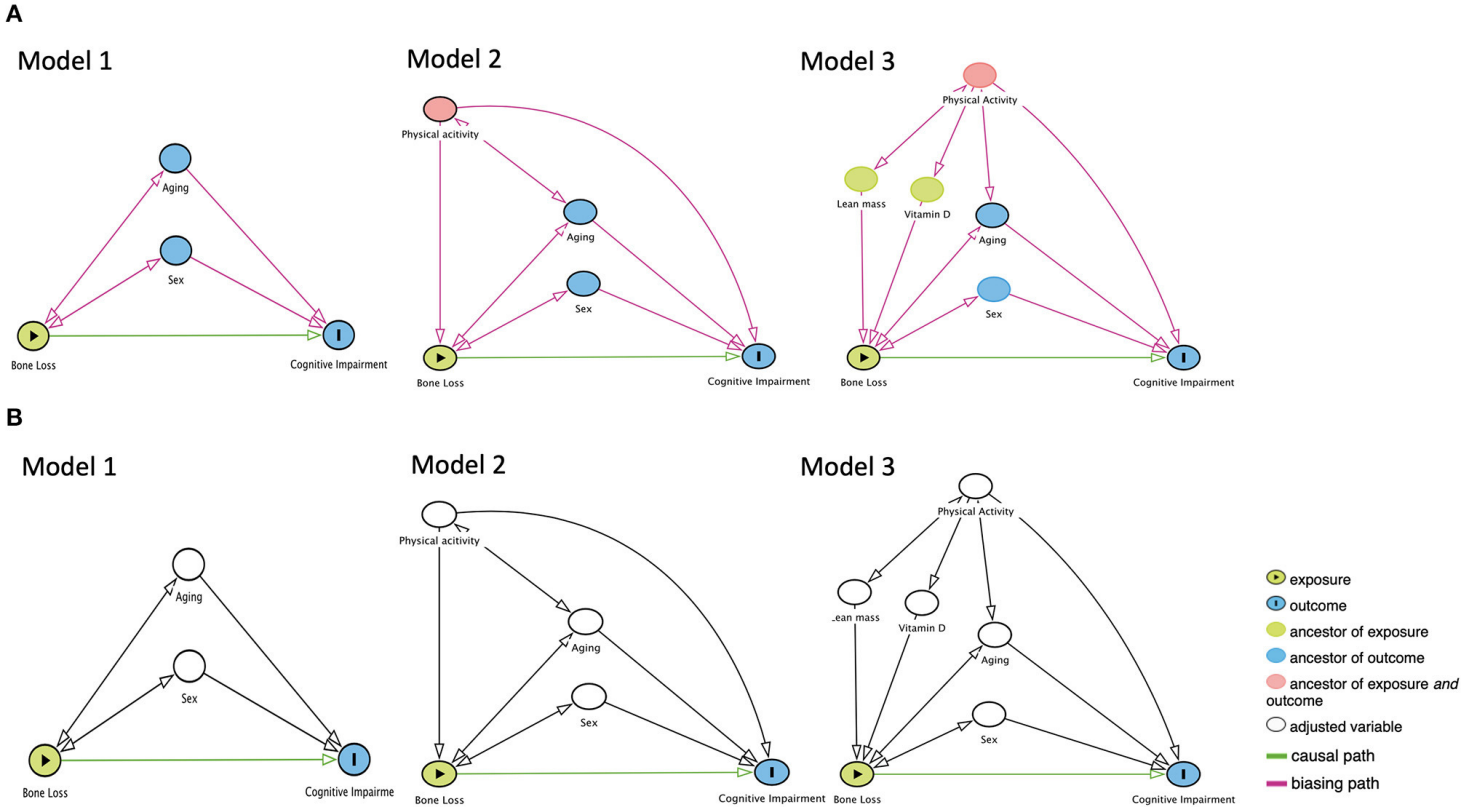
The proposed directed acyclic graphs between bone loss and cognitive impairment. **(A)** Models of the no adjustment of confounders. **(B)** Models of the adjustment of confounders.

The DAGs were plotted by background knowledge (Figure 1). In Model 1, the unbiased causal path between bone loss and cognitive impairment was plotted with adjustment of age and sex. In Model 2, the causal path was plotted with adjustment of age, sex, and physical activity. In Model 3, the causal path was plotted with adjustment of the age, sex, physical activity, vitamin D, and total lean mass. The DAGs were produced by the DAGitty version 3.0 software (University of Lübeck, Germany).

### Statistical Analysis

The continuous and discrete variables were analyzed through the ANOVA and chi-squared test, respectively. The *p*-trend for the three cognitive groups was estimated by the generalized linear models for continuous variables and the Cochran–Armitage trend test for the discrete variable. We used the DAGs to decide which covariates were adjusted and put them into the multivariable model for obtaining the unbiasing results. The generalized linear mixed models (GLMMs) with the random intercept and unstructured covariance matrix were applied, with the MMSE scores being dependent variables and with total BMD (unadjusted model); with total BMD, age, and sex (in Model 1); with total BMD, age, sex, and physical activity (in Model 2); or with total BMD, age, sex, physical activity, vitamin D, and total lean mass (in Model 3) being independent variables. The model fit statistics of the Akaike information criterion (AIC) and the Schwarz information criterion (SIC) were used to select the best model. For the AIC and the SIC, a lower score indicates a better model (34). The statistical significance was defined as two-tailed *p* < 0.05. All the statistical analyses were performed by using SAS 9.4 (Cary, North Carolina, USA). In the subgroup analysis by gender, the GLMMs with the above settings were applied. Required sample sizes for the subgroup analysis were estimated by specifying an α error of 0.05, a power (1-β) of 0.80, the number of covariates of 5, and the *R*^2^ values obtained in the unadjusted model.

### Validation Study

To examine the relationship between bone loss and cognitive impairment in Models 1 to 3, we employed the SEM. For explaining the SEM fit, we focused on the chi-squared test, comparative fit index (CFI), and standardized root mean square residual (SRMR). The chi-squared test for the SEM with *p* < 0.05 was defined as a good model fit. The values of the CFI and SRMR ≥ 0.90 and the SEM < 0.80 were considered as acceptable levels of fit, respectively (35–37). For the assessment of the direct effects, β coefficient of < 0.05 was considered to be unmeaningful, β coefficient of 0.05–0.09 was small but meaningful, β coefficient of 0.10–0.24 was moderate, and β coefficient of ≥ 0.25 was large. For the assessment of the indirect effects, β coefficient of < 0.003 was unmeaningful, β coefficient of 0.003–0.010 was small but meaningful, β coefficient of 0.010–0.060 was moderate, and β coefficient of ≥ 0.060 was large (38).

## Results

### Demographic Characteristics

**Figure 2:**
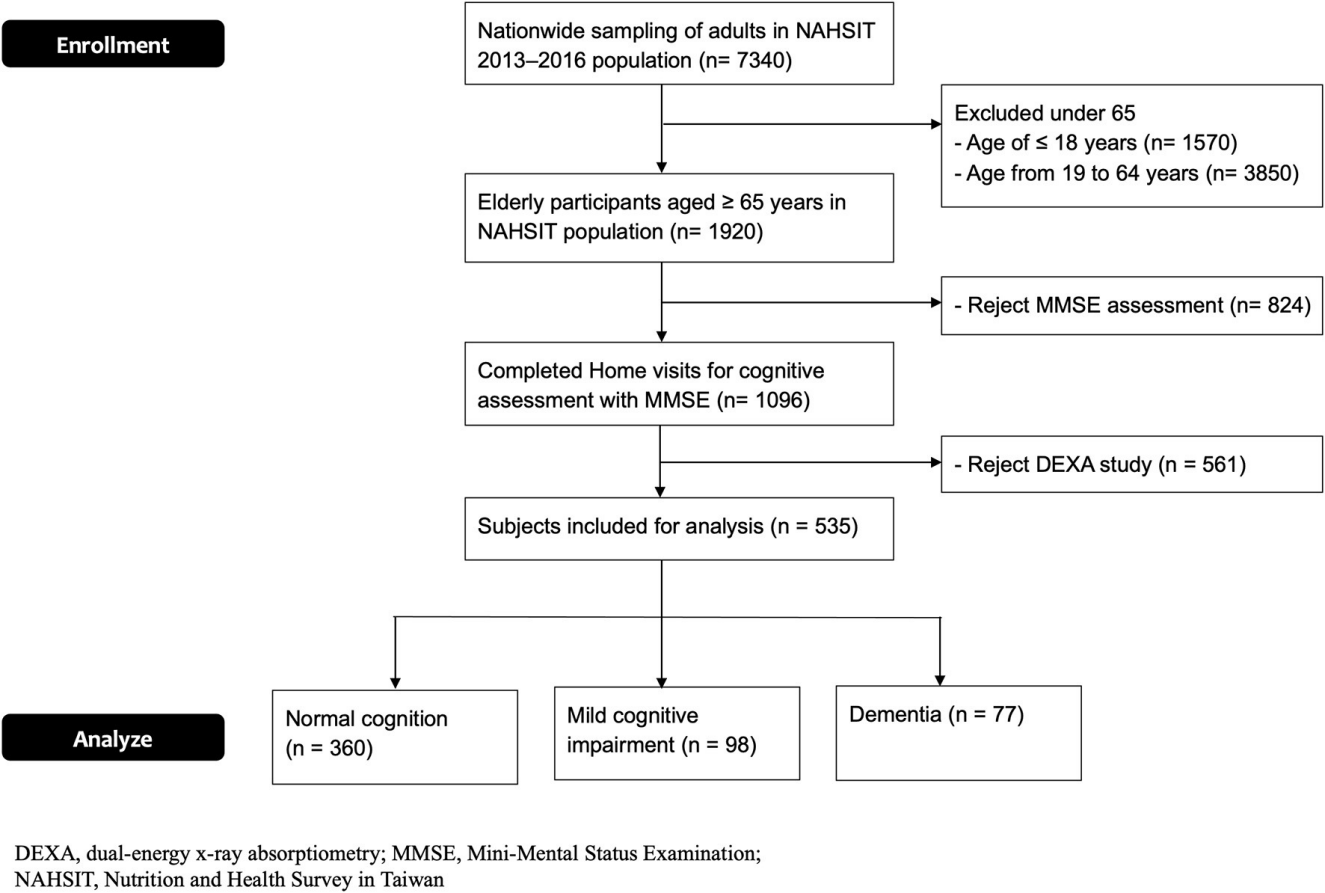
Flowchart of the study.

The flowchart of enrolling the elder participants in this cross-sectional study is shown in Figure 2. A total of 535 elder participants who completed the assessment were enrolled (Table 1). The average MMSE scores for the normal cognition, MCI, and dementia groups were 28.5 ± 1.2, 24.7 ± 1.0, and 12.8 ± 9.6 (*p* <0.0001, *p*-trend < 0.0001), respectively. In these three groups, the average ages were 71.3 ± 5.6, 73.2 ± 7.0, and 75.1 ± 6.8 (*p* < 0.0001, *p*-trend < 0.0001) and the education years were 9.5 ± 4.9, 8.2 ± 4.3, and 5.7 ± 4.2 (*p* < 0.0001, *p*-trend < 0.0001), respectively. Dementia and MCI groups had more female participants compared to the normal cognition. No significant difference in BMI was observed among these three groups.

**Table 1:** Demographic characteristics of the three cognitive groups (*N* = 535).

**Cognitive function**	**Normal cognition**	**Mild cognitive impairment**	**Dementia**	***P*** **value**	***P*** **trend**
Number (%)	360/535 (67.3%)	98/535 (18.3%)	77/535 (14.4%)		
MMSE score	28.5 ± 1.2	24.7 ± 1.0	12.8 ± 9.6	<0.0001^*^	<0.0001^*^
Age (years)	71.3 ± 5.6	73.2 ± 7.0	75.1 ± 6.8	<0.0001^*^	<0.0001^*^
65–69 years	164/360 (45.6%)	38/98 (38.8%)	19/77 (24.7%)	<0.0001^*^	
70–74 years	101/360 (28.1%)	22/98 (22.5%)	19/77 (24.7%)		
75–79 years	62/360 (17.2%)	19/98 (19.4%)	11/77 (14.3%)		
≥80 years	33/360 (9.2%)	19/98 (19.4%)	28/77 (36.4%)		
Female Sex	138/360 (38.3%)	45/98 (45.9%)	50/77 (64.9%)	<0.0001^*^	<0.0001^*^
BMI (kg/m^2^)	24.6 ± 3.5	24.9± 4.0	24.5 ± 3.8	0.6977	0.9175

* *Statistical significance (p < 0.05)*.

*BMI, body mass index; MMSE, Mini-Mental State Examination*.

### Bone Marrow Density and Osteoporosis

The measurements of BMD in the whole body and specific body regions for the three cognitive groups are shown in Table 2. With increasing in the cognitive impairment, the whole-body BMD revealed the decreasing values (1.102 ± 0.136, 1.096 ± 0.136, 1.013 ± 0.139 g/cm^2^, *p* < 0.0001, *p*-trend < 0.0001) among the normal cognition, MCI, dementia groups, respectively. Besides, BMD also showed the trend of decreasing values consistently in the upper extremities (*p* = 0.0005, *p*-trend = 0.0016), lower extremities (*p* < 0.0001, *p*-trend < 0.0001), spine (*p* < 0.0001, *p* trend < 0.0001), trunk area (*p* < 0.0001, *p*-trend < 0.0001), and femoral neck (*p* < 0.0001, *p*-trend < 0.0001), respectively. On the basis of the WHO definition, osteoporosis was more prevalent in the older adults with more severe cognitive impairment [normal cognition: 15.8%, MCI: 23.5%, and dementia groups: 32.5%, respectively (*p* = 0.0022, *p*-trend = 0.0005)]. The prevalence of osteoporosis in the overall participants was 19.6% (105/535).

**Table 2:** Dual-energy X-ray absorptiometry (DXA) measurements for bone marrow density in the three cognitive groups.

**Cognitive function**	**Normal cognition**	**Mild cognitive impairment**	**Dementia**	***P*** **value**	***P*** **trend**
**Bone marrow density (g/cm^2^)**					
Whole body	1.102 ± 0.136	1.096 ± 0.136	1.013 ± 0.139	<0.0001^*^	<0.0001^*^
Upper extremities	0.808 ± 0.157	0.813 ± 0.164	0.733 ± 0.136	0.0005^*^	0.0016^*^
Lower extremities	1.187 ± 0.178	1.170 ± 0.178	1.079 ± 0.191	<0.0001^*^	<0.0001^*^
Spine	1.064 ± 0.203	1.042 ± 0.214	0.947± 0.180	<0.0001^*^	<0.0001^*^
Trunk	0.880 ± 0.132	0.865 ± 0.143	0.797 ± 0.120	<0.0001^*^	<0.0001^*^
**Bone marrow density (g/cm** ^**2**^ **) by specific body region**					
Femoral neck	0.812 ± 0.150	0.786 ± 0.177	0.728 ± 0.147	<0.0001^*^	<0.0001^*^
L-spine (L1 L4)	1.053 ± 0.217	1.020 ± 0.192	0.958 ± 0.235	0.0520	0.0162^*^
**T-score**					
Femoral neck	−1.134 ± 1.228	−1.235 ± 1.314	−1.642 ± 0.999	0.0053^*^	0.0022^*^
L-spine (L1 to L4)	−0.590 ± 1.760	−0.843 ± 1.561	−1.346 ± 1.934	0.0598	0.0193^*^
Osteopenia (%)	72/360 (20.0%)	20/98 (20.4%)	14/77 (18.2%)	0.9237	0.7752
Osteoporosis (%)	57/360 (15.8%)	23/98 (23.5%)	25/77 (32.5%)	0.0022^*^	0.0005^*^

* *Statistical significance (p < 0.05)*.

*MMSE, Mini-Mental State Examination*.

### Body Composition, Physical Activity, and Laboratory Tests

For body composition parameters (Table 3), the total lean mass was decreasing significantly with increasing in the cognitive impairment (40.79 ± 7.38, 39.14 ± 7.38, 37.21 ± 7.17 kg, *p* = 0.0006, *p*-trend < 0.0001). The lean mass in the arms (*p* = 0.0047, *p*-trend = 0.0011), trunk (*p* = 0.0047, *p*-trend = 0.0011), android (*p* = 0.0045, *p*-trend = 0.0012), gynoid (*p* < 0.0001, *p*-trend < 0.0001), and legs (*p* = 0.0045, *p*-trend = 0.0012) revealed as the same trend as lean mass in whole body. For fat mass, the three cognitive groups showed no significant differences among them. For physical activity (Table 4), the MCI group showed a little higher activity, but no significant differences were found in the three groups. The laboratory tests of the lipid profiles, ferritin, vitamin A, and vitamin D showed no significant differences among the three cognitive groups as well (Table 4).

**Table 3:** Dual-energy X-ray absorptiometry measurements for body composition in the three cognitive groups.

**Cognitive function**	**Normal cognition**	**Mild cognitive impairment**	**Dementia**	***P*** **value**	***P*** **trend**
**Body composition of lean mass**					
Total (kg)	40.79 ± 7.38	39.14 ± 7.38	37.21 ± 7.17	0.0006^*^	<0.0001^*^
Arms (kg)	4.58 ± 1.12	4.37 ± 1.03	4.13 ± 1.10	0.0047^*^	0.0011^*^
Trunk (kg)	19.76 ± 3.48	19.14 ± 3.63	18.22 ± 3.38	0.0030^*^	0.0007^*^
Android (kg)	2.97 ± 0.61	2.88 ± 0.68	2.71 ± 0.60	0.0045^*^	0.0012^*^
Gynoid (kg)	6.12 ± 1.22	5.79 ± 1.27	5.41 ± 1.23	<0.0001^*^	<0.0001^*^
Legs (g)	13.20 ± 2.73	12.48 ± 2.77	11.83 ± 2.69	0.0003^*^	<0.0001^*^
**Body composition of fat mass**					
Total (kg)	19.67 ± 6.94	20.06 ± 6.88	19.18 ± 6.24	0.7201	0.7650
Arms (kg)	1.98 ± 0.84	2.09 ± 0.84	2.11 ± 0.84	0.3712	0.1741
Trunk (kg)	11.44 ± 4.30	11.51 ± 4.26	10.91 ± 3.90	0.6137	0.4494
Android (kg)	2.09 ± 0.84	2.14± 0.87	1.97 ± 0.77	0.4291	0.4945
Gynoid (kg)	3.09 ± 1.08	3.13 ± 1.04	3.08 ± 0.98	0.9364	0.9541
Legs (kg)	5.48 ± 2.18	5.70 ± 2.11	5.42 ± 1.96	0.6411	0.9189

* *Statistical significance (p < 0.05)*.

*MMSE, Mini-Mental State Examination*.

**Table 4:** Physical activity of the three cognitive groups.

**Cognitive function**	**Normal cognition**	**Mild cognitive impairment**	**Dementia**	***P*** **value**	***P*** **trend**
Physical activity (MET hours/week)	2.64 ± 11.01	3.19 ± 17.84	1.20 ± 5.94	0.7108	0.4941
**Physical activity range**				0.7815	
≥15.00 MET hours/week	17/360 (4.7%)	5/98 (5.1%)	3/77 (4.0%)		
7.50–14.9 MET hours/week	13/360 (3.6%)	2/98 (2.0%)	1/77 (2.0%)		
3.75–7.49 MET hours/week	6/360 (1.7%)	2/98 (2.0%)	0/77 (0%)		
<3.75MET hours/week	324/360 (90.0%)	89/98 (90.8%)	73/77 (94.0%)		
**Lipid profile**					
Total cholesterol (mg/dL)	187.9 ± 36.8	181.3 ± 29.9	179.4 ± 32.6	0.0739	0.0267^*^
LDL-C (mg/dL)	118.7 ± 33.1	115.6 ± 27.4	112.2 ± 28.6	0.2389	0.0905
HDL-C (mg/dL)	53.5 ± 15.8	51.3 ± 12.9	51.7 ± 14.0	0.3691	0.2088
Triglycerides (mg/dL)	122.6 ± 75.7	110.1 ± 45.3	113.2 ± 55.8	0.2214	0.1407
Ferritin (ng/mL)	235.8 ± 256.4	220.6 ± 161.3	202.9 ± 149.6	0.5086	0.2450
Vitamin A (μM)	2.3 ± 0.7	2.2± 0.8	2.3 ± 0.8	0.8295	0.7519
Vitamin D (μM)	34.3 ± 10.6	36.7 ± 11.7	32.9 ± 12.5	0.0821	0.7966

* *Statistical significance (p < 0.05)*.

*HDL-C, high density lipoprotein-cholesterol; LDL-C, low density lipoprotein-cholesterol; MET, metabolic equivalent of task*.

### Associations Between Bone Loss and Cognitive Impairment

The regression analysis of the GLMMs is presented in Table 5. In the unadjusted models, decrease of total BMD showed significantly decrease of the MMSE score (β = 8.479 per g/cm^2^ decrease, SE = 2.072, *p* < 0.0001). In Model 1, the significant association between total BMD and the MMSE score remained with adjustment of the age and sex (β = 5.792 per g/cm^2^ decrease, SE = 2.681, *p* = 0.0312). In Model 2, this significant association was persisted even if adjustment of the age, sex, and physical activity (β = 5.819 per g/cm^2^ decrease, SE = 2.682, *p* = 0.0305). In Model 3, no statistically significant association between the bone loss (β = 5.884 per g/cm^2^ decrease, SE = 3.092, *p* = 0.0576) and cognitive impairment was found with adjustment of the age, sex, physical activity, vitamin D, and total lean mass. In comparison to Models 1 and 2, Model 3 with the lowest values of the AIC (3014.11) and the SIC (3010.11) was considered the best model, which was furtherly used for the validation with an SEM.

**Table 5:** Generalized linear mixed model estimates of the Mini-Mental State Examination (MMSE).

**Scores of each item**	**Unadjusted**			**Model 1**			**Model 2**			**Model 3**		
**(*N* = 535)**	**Total MMSE Score^†^**			**Total MMSE Score^†^**			**Total MMSE Score^†^**			**Total MMSE Score^†^**		
	**β**	**S.E.**	***P*** **value**	**β**	**S.E.**	***P*** **value**	**β**	**S.E.**	***P*** **value**	**β**	**S.E.**	***P*** **value**
Total BMD (per g/cm^2^)	−8.479	2.072	<0.0001^*^	−5.792	2.681	0.0312^*^	−5.819	2.682	0.0305^*^	−5.884	3.092	0.0576
Age (per 5 years)				−0.768	0.242	0.0016^*^	−0.764	0.242	0.0017^*^	−0.772	0.248	0.0020^*^
Sex (female vs male)				−0.826	0.754	0.2741	−0.786	0.756	0.2989	−1.825	1.000	0.0686
Physical Activity (per MET hours/week)							0.019	0.024	0.4278	0.020	0.023	0.3939
Vitamin D										−0.051	0.028	0.0772
Total lean mass (per kg)										−0.075	0.071	0.0391^*^
**Model fit statistics**												
AIC	3398.72			3391.61			3396.64			3014.11		
SIC	3394.72			3387.61			3392.64			3010.11		

* *Variables with statistical significance (p < 0.05)*.

† *Regression analysis of the generalized linear mixed model with the random intercept (multilevel model) was employed*.

*AIC, Akaike information criterion; BMD, bone marrow density; MET, metabolic equivalent of the task; SIC, Schwarz information criterion*.

There was no sex difference observed in the GLMMs (Supplementary Table S1). In the unadjusted models, decrease of total BMD showed significantly decrease of the MMSE score in the males (β = 6.926 per g/cm^2^ decrease, SE = 2.986, *p* = 0.0211) and in the females (β = 9.857 per g/cm^2^ decrease, SE = 4.778, *p* = 0.0403), respectively. After adjustment for the age, physical activity, vitamin D, and total lean mass, a decrease of total BMD showed a reduction of the MMSE but without statistically significant. However, the sample size in the subgroup analysis by gender was inadequate (Supplementary Table S2). This study had 535 participants and out of them, 302 were males and 233 were females. The minimum sample size was 421 to attain an α error of 0.05 and apower (1-β) of 0.80 in the GLMMs with five covariates.

### Validation With Structural Equation Modeling

**Figure 3:**
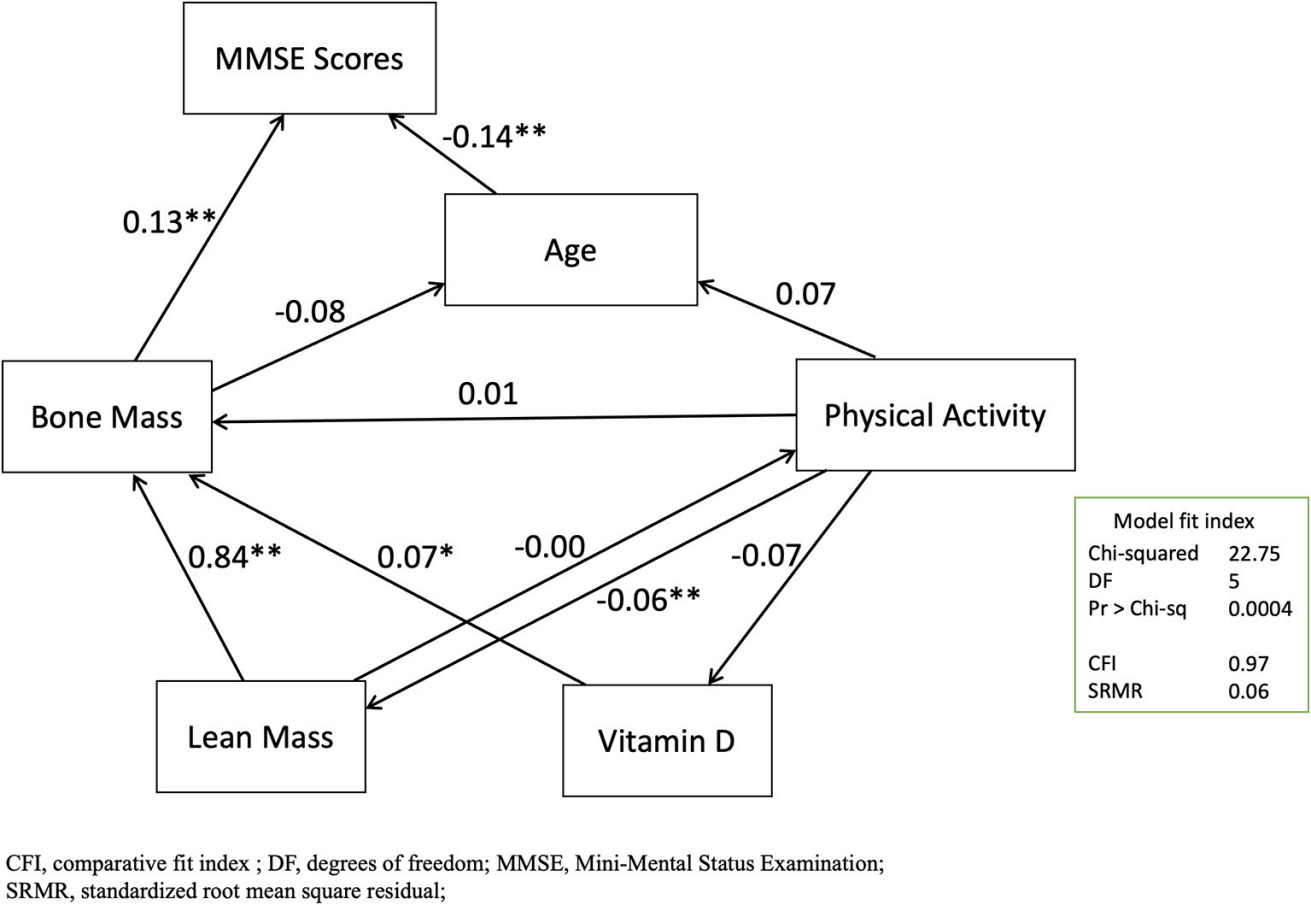
Graphical presentation of the structural equation modeling. ^*^p < 0.05, ^**^p < 0.01.

In this study, the final SEM for the variables of age, bone mass, lean mass, vitamin D, physical activity, and the MMSE scores were plotted (Figure 3). This model also showed acceptable levels of fit [chi-squared test 22.75 with degrees of freedom of 5 (*p* =0.0004), CFI: 0.97, adjusted CFI: 0.93, SRMR: 0.06]. The standardized effects for the direct, indirect, and total effects were listed (Supplementary Tables S3–S5). In the direct paths, the predictive model confirmed that the MMSE score was significantly and moderately predicted by age (β = 0.1363, *p* = 0.0003) and bone mass (β = 0.1251, *p* = 0.0006) by total BMD. In the indirect paths, the MMSE score was significantly and strongly predicted by lean mass (β = 0.1138, *p* = 0.0003).

## Discussion

This study examined the association between bone mass and cognitive function for ethnic Asian elder adults in Taiwan. We found that bone loss was independently associated with cognitive impairment in the primary analysis with the GLMMs. The association between bone mass and cognitive function was not simply the confounding effect of age or change of body composition. These findings were reproducible and confirmed by our separate validation study with SEM.

An earlier hospital-based study, using a nationwide health insurance database for the patients in Taiwan who were diagnosed as having osteoporosis and related fractures [the International Classification of Diseases-9-Clinical Modification (ICD-9-CM) codes 733.0 and 733.1], revealed a 1.4-fold relative risk of developing dementia (39). Another similar study conducted in Germany that analyzed patients who had osteoporosis-related fractures (the ICD-10 codes M80 and M81) from 1993 to 2012 revealed that the patients with osteoporosis had a 1.2–1.3-fold higher risk of developing dementia (6). This study extended and confirmed that the association between bone mass and cognitive impairment existed in both the hospital-based and community-based populations. In addition, the participants in this study were noninstitutionalized healthy elder adults. This indicated that bone health should be emphasized before elder adults developed obvious symptoms and signs of cognitive impairment.

Researchers have previously hypothesized that dementia and osteoporosis share mechanisms or are linked in other forms. In the SEM models, we found that the age and bone density individually directly predicted the MMSE score. This should be compatible with the unadjusted Model 1 and Model 2 in the regression analyses with the GLMMs. Therefore, we inferred that age and BMD were independently associated with cognitive impairment. On the other hand, BMD was not significantly associated with the MMSE score in Model 3 with adjustment of the vitamin D and lean mass. Additionally, lean mass was found significantly associated with the MMSE score in Model 3. In the SEM models, we found that the MMSE score was strongly and indirectly predicted by lean mass. These indirect paths from the lean mass to bone density, in the negative sense, should explain the attenuated association between BMD and the MMSE score by the GLMMs in Model 3. Additionally, we determined the SEM with acceptable fit with the three indicators of chi-squared test, CFI, and SRMR. Despite the root mean square error of approximation (RMSEA) was commonly used, we have the three main reasons that prefer SRMR to RMSEA: (1) SRMR is more accurate than RMSEA across small-to-large sample sizes (36, 40), (2) SRMR produces less type I error than RMSEA (36), and (3) RMSEA is more likely to overreject the true population models by using the proposed cutoff criteria (41). Thus, we determined to use the three indicators, including the chi-squared test, CFI, and SRMR, to have an overall judgment of the good model fit.

The representative sampling of the community-based elder participants in this study should also be our strength. Proportions or prevalence of normal cognition, MCI, and dementia were similar to another nationwide population-based cross-sectional survey of cognitive impairment in Taiwan (42, 43). This study also had some limitations. First, owing to the cross-sectional design, we could not directly obtain the causal relationship between bone loss and cognitive impairment. However, with the SEM models, we may infer the most reasonable causal paths between bone loss and cognitive impairment. Second, the NAHSIT study focused on surveying the nutritional status of the elder participants and contained no more advanced genetic biomarkers. Third, this study had inadequate sample sizes in the subgroup analysis by gender. Though the overall analysis exhibited consistent results in both the males and females (Supplementary Table S1), larger sample size was required to confidently conclude no gender difference between the bone loss and cognitive impairment (Supplementary Table S2). Fourth, since this study aimed to survey the health status in the community population, the participants with higher health awareness were with higher willingness to complete all the assessments. A healthy volunteer bias possibly occurred and, therefore, the association between bone loss and cognitive impairment could be underestimated in this study. While the prevalence of osteoporosis in this study was 19.6%, the prevalence of osteoporosis by the National Health Insurance Research Database (NHIRD) of Taiwan, a real-world health database with coverage of > 99.9% residents in Taiwan (44), was from 17.4 to 25.0% (32). Our prevalence of MCI and dementia was similar to the prevalence by the NHIRD of Taiwan (42). Additionally, to ensure the representativeness for the total population in Taiwan, the NAHSIT 2013–2016 adopted the stratified sampling design by the characteristics of population density (with consideration of age and sex distribution) and geographical area. The enrollment protocol of the latest NAHSIT 2013–2016 (45) and the previous NAHSIT 2005–2008 (46) has been previously published. Consequently, we considered that the representative of the enrolled participants was not threatened.

In conclusion, these results support the association between bone loss and cognitive impairment for the older adults that were present and not simply a confounding effect from aging. The decrease of lean mass may indirectly affect cognitive impairment by influencing bone density. Further studies focus on exploring the biological plausibility that should be more convincing.

## Data Availability Statement

The datasets presented in this article are not readily available because the NAHSIT 2013–2016 study was managed by the Health Promotion and Administration (HPA), Ministry of Health and Welfare, Taiwan. With legal restrictions imposed by the government of Taiwan on the distribution of the personal health data in relation to the Personal Information Protection Act, request for data needs a formal proposal to the Health and Welfare Data Science Center (HWDC), Ministry of Health and Welfare, Taiwan, and therefore the data was not publicly available. Requests to access the datasets should be directed to Ministry of Health and Welfare, Taiwan, https://www.mohw.gov.tw/np-126-2.html.

## Ethics Statement

The studies involving human participants were reviewed and approved by Institutional Review Board on Biomedical Science Research, Academia Sinica, Taiwan; Research Ethics Committee, National Health Research Institutes, Taiwan. The patients/participants provided their written informed consent to participate in this study.

## Author Contributions

S-FL, Y-CF, W-HP, and C-HB contributed to the conception and design of the study, acquisition, analysis, and interpretation of the data. S-FL wrote the first draft of the article. All authors contributed to the article and approved the submitted version.

## Funding

This study was funded by the Health Promotion Administration (HPA), Ministry of Health and Welfare, Taiwan (reference number: MOHW108-HPAH-114-134703 and MOHW109-HPA-H-114-144702), and by the Ministry of Science and Technology, Taiwan, in the form of a grant awarded to CHB (reference number: MOST 107-2314-B-038-072-MY3 and MOST 110-2314-B038-056-MY3). The content of this research may not represent the opinion of the Health Promotion Administration, Ministry of Health and Welfare, Taiwan.

## Conflict of Interest

The authors declare that the research was conducted in the absence of any commercial or financial relationships that could be construed as a potential conflict of interest.

## Publisher's Note

All claims expressed in this article are solely those of the authors and do not necessarily represent those of their affiliated organizations, or those of the publisher, the editors and the reviewers. Any product that may be evaluated in this article, or claim that may be made by its manufacturer, is not guaranteed or endorsed by the publisher.
