# Corrigendum: Bone and Lean Mass Loss and Cognitive Impairment for Healthy Elder Adults: Analysis of the Nutrition and Health Survey in Taiwan 2013–2016 and a Validation Study With Structural Equation Modeling

**DOI:** 10.3389/fnut.2021.839017

**Published:** 2022-04-29

**Authors:** Sheng-Feng Lin, Yen-Chun Fan, Wen-Harn Pan, Chyi-Huey Bai

**Affiliations:** ^1^Department of Public Health, School of Medicine, College of Medicine, Taipei Medical University, Taipei, Taiwan; ^2^School of Public Health, College of Public Health, Taipei Medical University, Taipei, Taiwan; ^3^Department of Emergency Medicine, Taipei Medical University Hospital, Taipei, Taiwan; ^4^Department of Critical Care Medicine, Taipei Medical University Hospital, Taipei, Taiwan; ^5^Institute of Biomedical Sciences, Academia Sinica, Taipei, Taiwan; ^6^Nutrition Research Center, Taipei Medical University Hospital, Taipei, Taiwan

**Keywords:** bone loss, bone marrow density, cognitive impairment, dementia, osteoporosis

In the published article, there were errors in affiliations 1–6. Instead of “1 School of Public Health, College of Public Health, Taipei Medical University, Taipei, Taiwan. 2 Department of Critical Care Medicine, Taipei Medical University Hospital, Taipei, Taiwan. 3 Department of Clinical Pathology, Taipei Medical University Hospital, Taipei, Taiwan. 4 Institute of Biomedical Sciences, Academia Sinica, Taipei, Taiwan. 5 Department of Public Health, College of Medicine, Taipei Medical University, Taipei, Taiwan. 6 Nutrition Research Center, Taipei Medical University Hospital, Taipei, Taiwan.” it should be “1 Department of Public Health, School of Medicine, College of Medicine, Taipei Medical University, Taipei, Taiwan. 2 School of Public Health, College of Public Health, Taipei Medical University, Taipei, Taiwan. 3 Department of Emergency Medicine, Taipei Medical University Hospital, Taipei, Taiwan. 4 Department of Critical Care Medicine, Taipei Medical University Hospital, Taipei, Taiwan. 5 Institute of Biomedical Sciences, Academia Sinica, Taipei, Taiwan. 6 Nutrition Research Center, Taipei Medical University Hospital, Taipei, Taiwan.”

The authors apologize for this error and state that this does not change the scientific conclusions of the article in any way. The original article has been updated.

## Publisher's Note

All claims expressed in this article are solely those of the authors and do not necessarily represent those of their affiliated organizations, or those of the publisher, the editors and the reviewers. Any product that may be evaluated in this article, or claim that may be made by its manufacturer, is not guaranteed or endorsed by the publisher.

