# Supplementary material for: Bone and Lean Mass Loss and Cognitive Impairment for Healthy Elder Adults: Analysis of the Nutrition and Health Survey in Taiwan 2013–2016 and a Validation Study With Structural Equation Modeling
Source: Front Nutr. 2021 Oct 13;8:747877. doi: 10.3389/fnut.2021.747877 (PMC8548616; doi:10.3389/fnut.2021.747877)
Supplement: Supplementary file 1 [file Data_Sheet_1.PDF]

Supplemental Tables.

Number of Tables: 5

Table S1. Generalized Linear Mixed Model Estimates of Mini-mental Status Examination in Men and Women

Scores of each item	Unadjusted Total MMSE Score†			Model 1 Total MMSE Score†			Model 2 Total MMSE Score†			Model 3 Total MMSE Score†		
	β	S.E.	*P* value	β	S.E.	*P* value	β	S.E.	*P* value	β	S.E.	*P* value
Men (*N* = 302)												
Total BMD (per g/cm^2)	-6.926	2.986	0.0211*	-4.989	3.083	0.1067	-5.013	3.086	0.1054	-4.830	3.648	0.1867
Age (per 5 years)				-0.622	0.271	0.0266*	-0.622	0.272	0.0228*	-0.528	0.297	0.0770
Physical Activity (per MET hours/week)							0.016	0.024	0.5078	0.015	0.025	0.5425
Vitamin D (μM)										-0.035	0.033	0.2911
Total lean mass (per kg)										-0.050	0.081	0.5394
Women (*N* = 233)												
Total BMD (per g/cm^2)	-9.857	4.778	0.0403*	-7.219	4.867	0.1394	-7.249	4.876	0.1386	-7.129	5.618	0.2060
Age (per 5 years)				-1.062	0.458	0.0215*	-1.045	0.461	0.0243*	-1.199	0.449	0.0082*
Physical Activity (per MET hours/week)							0.024	0.055	0.6654	0.030	0.052	0.5644
Vitamin D (μM)										-0.079	0.049	0.1076
Total lean mass (per kg)										-0.077	0.144	0.5908
Model fit statistics												
AIC for men	1876.72			1875.49			1880.64			1722.61		
SIC for men	1872.72			1871.73			1876.64			1718.61		
AIC for women	1508.97			1506.59			1510.37			1296.60		
SIC for women	1504.97			1502.59			1506.37			1292.60		

BMD, bone marrow density; MET, metabolic equivalents; MMSE, Mini-Mental Status Examination. S.E., standard error.

*Variables with statistical significance ($P < 0.05$).

†Regression analysis of generalized linear mixed model with the random intercept (multilevel model) was employ

Table S2. Required Sample Size Estimation between Bone Loss and Cognitive Deficits

α value	Power $(1-\beta)$	Number of covariates	R^2	Estimated Required Number
0.05	0.80	5	0.18	706
0.05	0.80	5	0.19	668
0.05	0.80	5	0.20	635
0.05	0.80	5	0.21	604
0.05	0.80	5	0.22	576
0.05	0.80	5	0.23	551
0.05	0.80	5	0.24	528
0.05	0.80	5	0.25	506
0.05	0.80	5	0.26	486
0.05	0.80	5	0.27	468
0.05	0.80	5	0.28	451
0.05	0.80	5	0.29	436
0.05	0.80	5	0.30	421

Table S3. The Standardized Direct Effects for Path List.

	Age	Bone mass	Lean mass	Physical activity	Vitamin D
MMSE score	−0.1364**	0.1251**	-	-	-
Age	-	−0.0819	-	0.0667	-
Bone mass	-	-	0.8354***	0.0093	0.0652*
Lean mass	-	-	-	−0.0643***	-
Physical activity	-	-	−0.0007	-	-
Vitamin D	-	-	-	−0.0651	-

*$P < 0.05$, **$P < 0.01$, ***$P < 0.001$.

Table S4. The Standardized Indirect Effects for Path List.

	Age	Bone mass	Lean mass	Physical activity	Vitamin D
MMSE score	-	0.0112	0.1138**	−0.0157	0.0089
Age	-	-	−0.0684	0.0040	−0.0053
Bone mass	-	-	<0.0001	−0.0580***	-
Lean mass	-	-	<0.0001	<−0.0001	
Physical activity	-	-	<−0.0001	<0.0001	
Vitamin D	-	-	<0.0001	<−0.0001	

*$P < 0.05$, **$P < 0.01$, ***$P < 0.001$.

Table S5. The Standardized Total Effects for Path List.

	Age	Bone mass	Lean mass	Physical activity	Vitamin D
MMSE score	−0.1364**	0.1363**	0.1138**	−0.0157	0.0089
Age	-	−0.0819	−0.0684	0.0706	−0.0053
Bone mass	-	-	0.8355***	−0.0487	0.0652*
Lean mass	-	-	<0.0001	−0.0643***	-
Physical activity	-	-	−0.0007	<0.0001	-
Vitamin D	-	-	<0.0001	−0.0651	-

*$P < 0.05$, **$P < 0.01$, ***$P < 0.001$.
